# Mechanochemical Knoevenagel condensation investigated in situ

**DOI:** 10.3762/bjoc.13.197

**Published:** 2017-09-26

**Authors:** Sebastian Haferkamp, Franziska Fischer, Werner Kraus, Franziska Emmerling

**Affiliations:** 1 BAM Federal Institute for Materials Research and Testing, Richard-Willstaetter-Str. 11, 12489 Berlin, Germany; 2 Department of Chemistry, Humboldt-Universität zu Berlin, Brook-Taylor-Str. 2, 12489 Berlin, Germany

**Keywords:** ball milling, C–C coupling, in situ, Knoevenagel condensation, mechanochemistry

## Abstract

The mechanochemical Knoevenagel condensation of malononitrile with *p*-nitrobenzaldehyde was studied in situ using a tandem approach. X-ray diffraction and Raman spectroscopy were combined to yield time-resolved information on the milling process. Under solvent-free conditions, the reaction leads to a quantitative conversion to *p*-nitrobenzylidenemalononitrile within 50 minutes. The in situ data indicate that the process is fast and proceeds under a direct conversion. After stopping the milling process, the reaction continues until complete conversion. The continuous and the stopped milling process both result in crystalline products suitable for single crystal X-ray diffraction.

## Introduction

Mechanochemical syntheses have gained increasing popularity in different areas such as materials science, chemistry, and pharmacy. Especially for organic syntheses, mechanochemistry is currently implemented as a green, fast, and efficient synthesis approach [1–3]. The syntheses are either solvent-free or require only a minimum amount of solvent. Consequently, solvation and desolvation phenomena can be neglected [4–6]. Mechanochemical syntheses of organic systems provide for example an efficient method for cocrystal screening [7–9], an increased product selectivity [2,10–12], and a pathway to new products, which are inaccessible via traditional methods [3,13–14]. Often, stoichiometric reactions with quantitative yields of the final product are possible, rendering the use of solvents and work-up procedures unnecessary [3,15]. The mechanochemical synthesis can effect carbon–carbon and carbon–heteroatom covalent bonds, coordinating bonds between metal and ligands, and non-covalent interactions such as hydrogen bonds, halogen bonds, and π∙∙∙π interactions [16–19]. For example, Toda et al. reported yields of 97% for Aldol condensations in the absence of any solvents [20]. Kaupp et al. described the first Knoevenagel condensation in a ball mill [21]. Compared to conventional synthesis in which bases or Lewis acids are used as catalysts, Kaupp et al. could reduce the amount of catalysts [21]. The Knoevenagel condensation of *p*-nitrobenzaldehyde with malononitrile was initially only accessible in melts at 150–170 °C or in the presence of a catalyst like calcite or fluorite [21–22]. In an extended study, including different aldehydes, Ondruschka et al. reported a solvent- and catalyst-free Knoevenagel condensation of *p*-nitrobenzaldehyde and malononitrile in a vibrational mill [23]. Here, we report the first direct in situ investigation of a Knoevenagel condensation followed by combined X-ray diffraction and Raman spectroscopy measurements in a tandem approach. Our investigations reveal that the formation of the crystalline product begins after 36 min and is completed after 50 min. The reaction can be described as a melt-mediated reaction since malononitrile melts during the grinding process, whereas *p*-nitrobenzaldehyde remains crystalline until the onset of the product formation. The crystalline product was of sufficient quality for single crystal X-ray structure determination.

## Results and Discussion

**Scheme 1:**
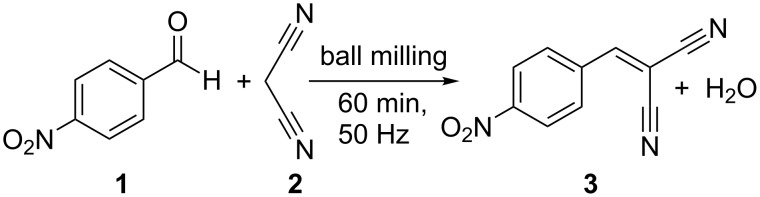
Knoevenagel condensation of *p*-nitrobenzaldehyde (**1**) with malononitrile (**2**) yielding *p*-nitrobenzylidenemalononitrile (**3**).

**Figure 1:**
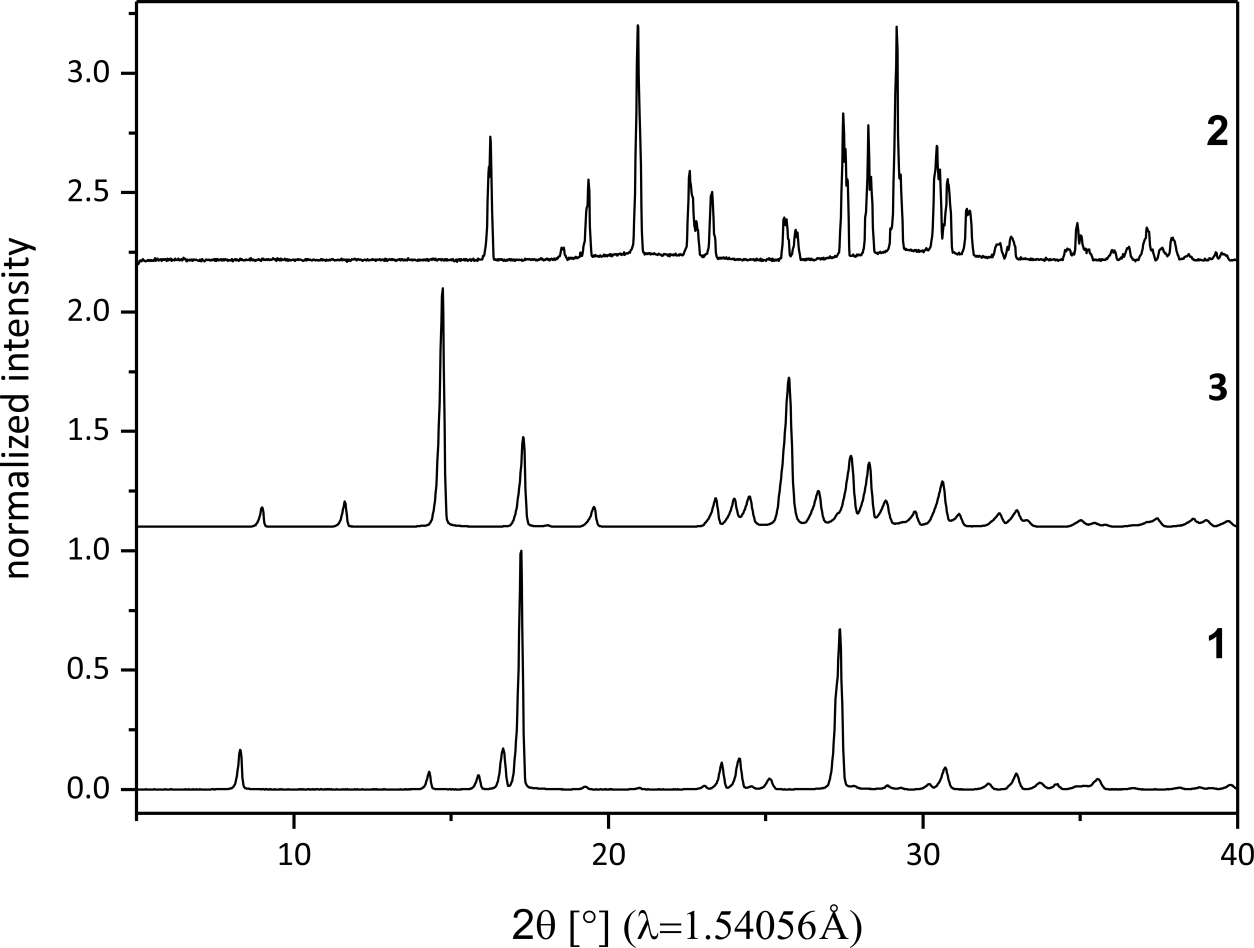
X-ray diffraction patterns of the reactants *p*-nitrobenzaldehyde (**1**) and malononitrile (**2**) and the product *p*-nitrobenzylidenemalononitrile (**3**).

Scheme 1 illustrates the investigated Knoevenagel condensation of *p*-nitrobenzaldehyde (**1**) with malononitrile (**2**) using a ball mill. The stoichiometric reaction mixture was ball-milled for 60 minutes at 50 Hz in a conventional ball mill with either stainless steel or Perspex grinding jars. For both jar materials, the X-ray diffraction patterns of the product in comparison to those of the reactants reveal a complete and quantitative reaction (see Figure 1). For the in situ investigations, time-resolved X-ray diffraction patterns and Raman spectra were recorded during the milling reaction. The Raman laser and the X-ray beam were focused on the same spot at the inner wall of a Perspex grinding jar, allowing to monitor the course of the reaction simultaneously. A detailed description of the experimental setup can be found elsewhere [24].

**Figure 2:**
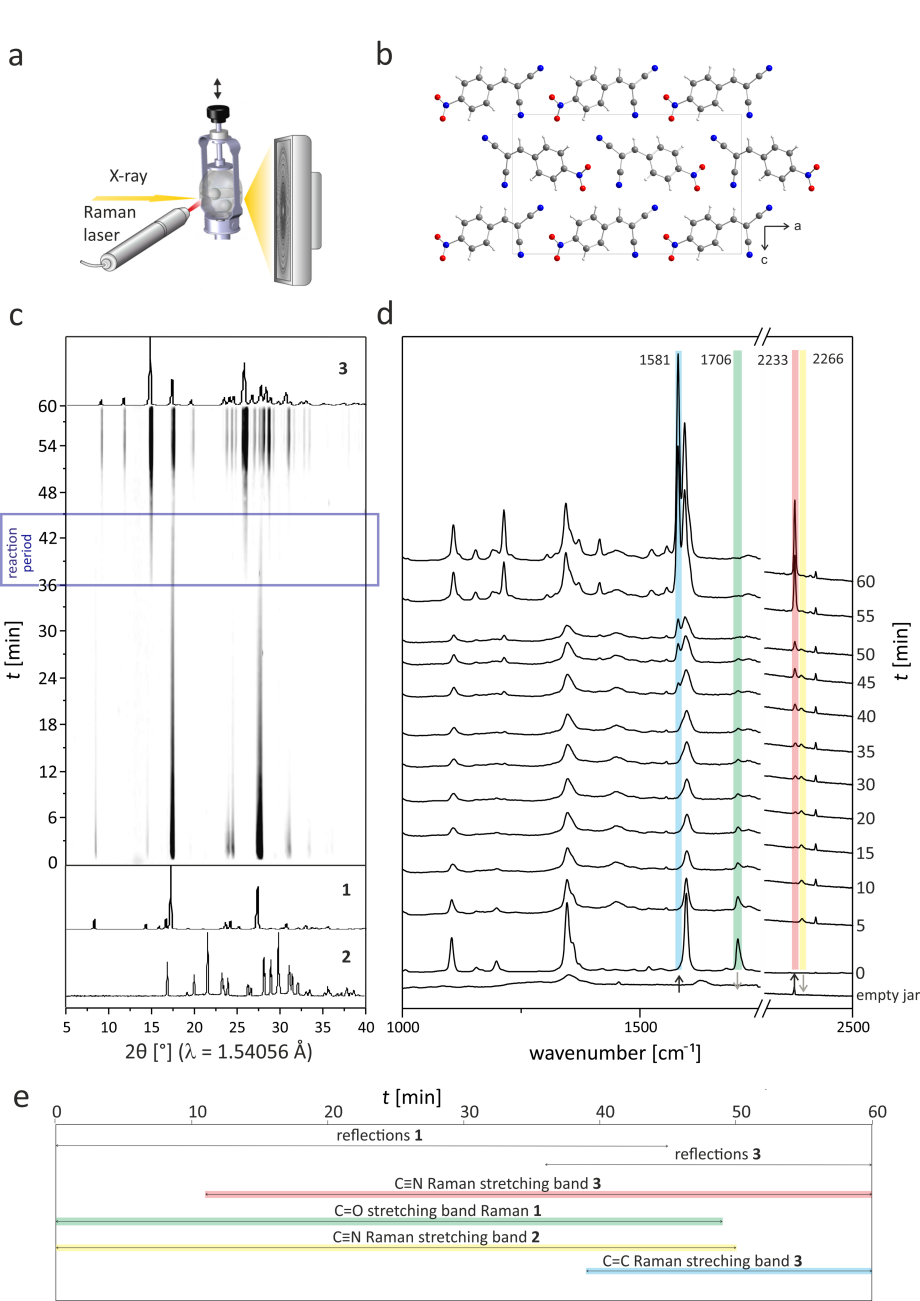
a) Schematic diagram of the in situ setup for investigating mechanochemical reactions in a tandem approach based on synchrotron X-ray powder diffraction and Raman spectroscopy. b) Crystal structure of the final product *p*-nitrobenzylidenemalononitrile (**3**) along the *b*-axis. c) Time-resolved X-ray diffraction patterns recorded during the Knoevenagel condensation of *p*-nitrobenzaldehyde with malononitrile yielding **3**. Three phases can be distinguished based on the XRPD data. During the first 36 minutes, the reflections of the reactant **1** are observed. Within a time-span of nine minutes, the product is formed, evident from two strong reflections at 2θ = 14.6° and 25.7°. d) Time-resolved Raman spectra measured simultaneously (Here, the Raman spectra are shown with a 5 min interval. The complete set of spectra is shown in Supporting Information File 1, Figure S1.) The progress of the reaction can be detected from the decreasing signal of the C=O stretching band of **1** at 1706 cm^−1^ (green) and the increasing signal of the C=C stretching band at 1581 cm^−1^ (blue). The band attributed to the C≡N stretching shifts from 2266 cm^−1^ (yellow) in **2** to 2233 cm^−1^ (red) in the product. The first Raman spectrum shows the contribution of the empty jar. e) Course of the reaction detected by XRPD and Raman spectroscopy.

Figure 2 shows the X-ray powder diffraction (XRPD) patterns and the Raman spectra of the in situ investigation, monitored in a time-span of 60 minutes. No reflections of **2** are observed during the reaction. Most probably, **2** melts directly after the start of the reaction. Based on previous thermography investigations [25], we can assume that the temperature in the milling jar rises quickly to 35 °C which is above the melting point of **2** (32 °C). The intensity of the **1** reflections decreases continuously and vanishes after 45 minutes. After 36 minutes, reflections of the products can be detected next to those of **1**. After 45 minutes, only the product reflections can be observed in the time-resolved XRPD patterns (see Supporting Information File 1, Figure S2 for a quantitiative evaluation of the XRPD data). The in situ Raman data show a decreasing signal of the C=O stretching band at 1706 cm^−1^ (see Figure 2, green box) and an increase of the C=C stretching band at 1581 cm^−1^ (see Figure 2, blue box). The C=C stretching band is shifted to lower wavenumbers due to a higher conjugation of π-electrons. The extended conjugation is also responsible for the shift of the C≡N stretching band from 2266 to 2233 cm^−1^ (see Figure 2, yellow and red box). The signal at 2233 cm^−1^ can be observed after 11 minutes of reaction, indicating that the first *p*-nitrobenzylidenemalononitrile molecules are formed in the condensation reaction. This can be also deduced from the decreasing intensity of the C=O stretching band of **1** at 1706 cm^−1^. The C=C stretching band of the product at 1581 cm^−1^ is first observed after 39 minutes. In accordance to the diffraction data, the evaluation of the Raman spectra indicates the completion of the reaction after 50 minutes. In the following, the Raman bands become narrower, signifying an increasing crystallinity of the product.

Since the in situ data reveal an onset of the reaction after 36 min, we investigated in further experiments whether ball milling is needed for the completion of the reaction. The ball mill was stopped after 36 minutes and the reaction in the closed jar was monitored with Raman spectroscopy. The data show that under these conditions the reaction to the final product is completed within two hours. Consequently, prolonged milling after the initiation of the reaction is not necessary for a complete conversion to the product, but accelerates the reaction. The XRPD patterns of the product for both types of experiments are comparable and the crystals obtained in the in situ reactions are of sufficient quality for X-ray single crystal determination. The crystal structure could be solved, matching the parameters described in the literature [26].

## Conclusion

Using an in situ tandem approach combining synchrotron X-ray powder diffraction and Raman spectroscopy, we investigated the mechanochemical Knoevenagel condensation of malononitrile with *p*-nitrobenzaldehyde in situ. The data show that once the reaction is initiated mechanochemically it proceeds via a direct conversion leading to a highly crystalline material. We could reveal that the activated reaction proceeds also without further milling. The in situ investigation of mechanochemical processes proved to be beneficial for optimizing the milling reactions.

## Experimental

**Materials:** All chemicals were used without further purification.

**Syntheses:** The milling experiments were performed in a commercial ball mill (Pulverisette 23, Fritsch, Germany). In a typical experiment, equimolar quantities of the reactants *p*-nitrobenzaldehyde (800 mg, 5.29 mmol) and malononitrile 349.67 mg, 5.29 mmol) were weighed in milling jars (10 mL, stainless steel or Perspex). Two milling balls (stainless steel, 4 g, 10 mm diameter) were added to the reaction mixture. The reaction was performed in two different setups: i) The samples were prepared in Perspex grinding jars for the in situ measurements. Either the tandem in situ combination (synchrotron XRPD combined with Raman spectroscopy) or in situ Raman spectroscopy was employed. The reactants were milled at 50 Hz for 60 minutes. ii) Alternatively, the reaction mixture was milled in stainless steel jars at 50 Hz for 45 minutes. The final products were characterized by XRPD.

**X-ray powder diffraction:** All samples were characterised by XRPD analysis using a Bruker D8 diffractometer: Cu Kα_1_ radiation (λ = 1.54106 Å), 5.0° ≤ 2 θ ≤ 60°. All data were obtained in transmission mode with an acquisition time of 3 s per step (step size 0.009°)

**In situ investigations:** The tandem in situ experiments were performed at the µspot beamline (BESSY II, Helmholtz Centre Berlin for Materials and Energy) [27]. For these experiments, a commercial ball mill (Pulverisette 23, Fritsch, Germany) equipped with a Perspex grinding jar was used, providing the necessary strength and transparency for the experiments. The diffraction experiments were performed at an energy of 12.4 keV and a wavelength of 1.0003 Å. The scattered intensities were recorded using a two-dimensional X-ray detector (MarMosaic, CCD 3072 × 3072). The scattering images were processed with FIT2D [28].

A Raman RXN1^TM^ analyzer (Kaiser Optical systems, France) was used for the Raman spectroscopy measurements. A non-contact probe head with a working distance of 6 cm and a spot size of 1 mm. The excitation wavelength was 785 nm. A typical measurement consists of five accumulated recordings for 5 s. A new measurement was started every 30 s.

**Single crystal X-ray diffraction:** Single crystal XRD measurements were performed on a D8 Venture diffractometer (Bruker AXS, Germany) using Mo Kα radiation (λ = 0.71073 Å) monochromatized by a graphite crystal. The crystals were measured at 150 K. Data reduction was performed with Bruker AXS SAINT and SADABS packages. The structure was solved by direct methods and refined by full-matrix least-squares calculation [29]. Anisotropic thermal parameters were employed for non-hydrogen atoms. The hydrogen atoms were treated isotropically with U_iso_ = 1.2 times the U_eq_ value of the parent atom. Crystal data: chemical formula C_10_H_5_N_3_O_2_, formula weight 199.17, orthorhombic, space group *Pna*2_1_, *a* = 19.4857(8) Å, *b* = 3.78060(10) Å, *c* =11.9120(5) Å, *V* = 877.53(6) Å^3^, Z = 4, *T* = 150(2) K, µ = 0.110 mm^−1^, 25807 reflections measured, 2366 unique reflections, 2222 observed reflections [I > 2σ(I)], R_1_obs = 0.0395, wR_2_obs = 0.0956. Crystal size: 0.38 × 0.28 × 0.02 mm.

## Supporting Information

File 1 Raman spectra and XRPD data.
