# Supplementary material for: Mechanochemical Knoevenagel condensation investigated in situ
Source: Beilstein J Org Chem. 2017 Sep 26;13:2010–4. doi: 10.3762/bjoc.13.197 (PMC5629385; doi:10.3762/bjoc.13.197)
Supplement: File 1 — Raman spectra and XRPD data. [file Beilstein_J_Org_Chem-13-2010-s001.pdf]

Supporting Information

for

Mechanochemical Knoevenagel condensation investigated in situ

Sebastian Haferkamp[1,2], Franziska Fischer[1], Werner Kraus[1] and Franziska Emmerling*[1]

Address: [1]BAM Federal Institute for Materials Research and Testing, Richard-Willstaetter-Str. 11, 12489 Berlin, Germany and [2]Department of Chemistry, Humboldt-Universität zu Berlin, Brook-Taylor-Str. 2, 12489 Berlin, Germany

Email: Franziska Emmerling - franziska.emmerling@bam.de

*Correspomding author

Raman spectra and XRPD data

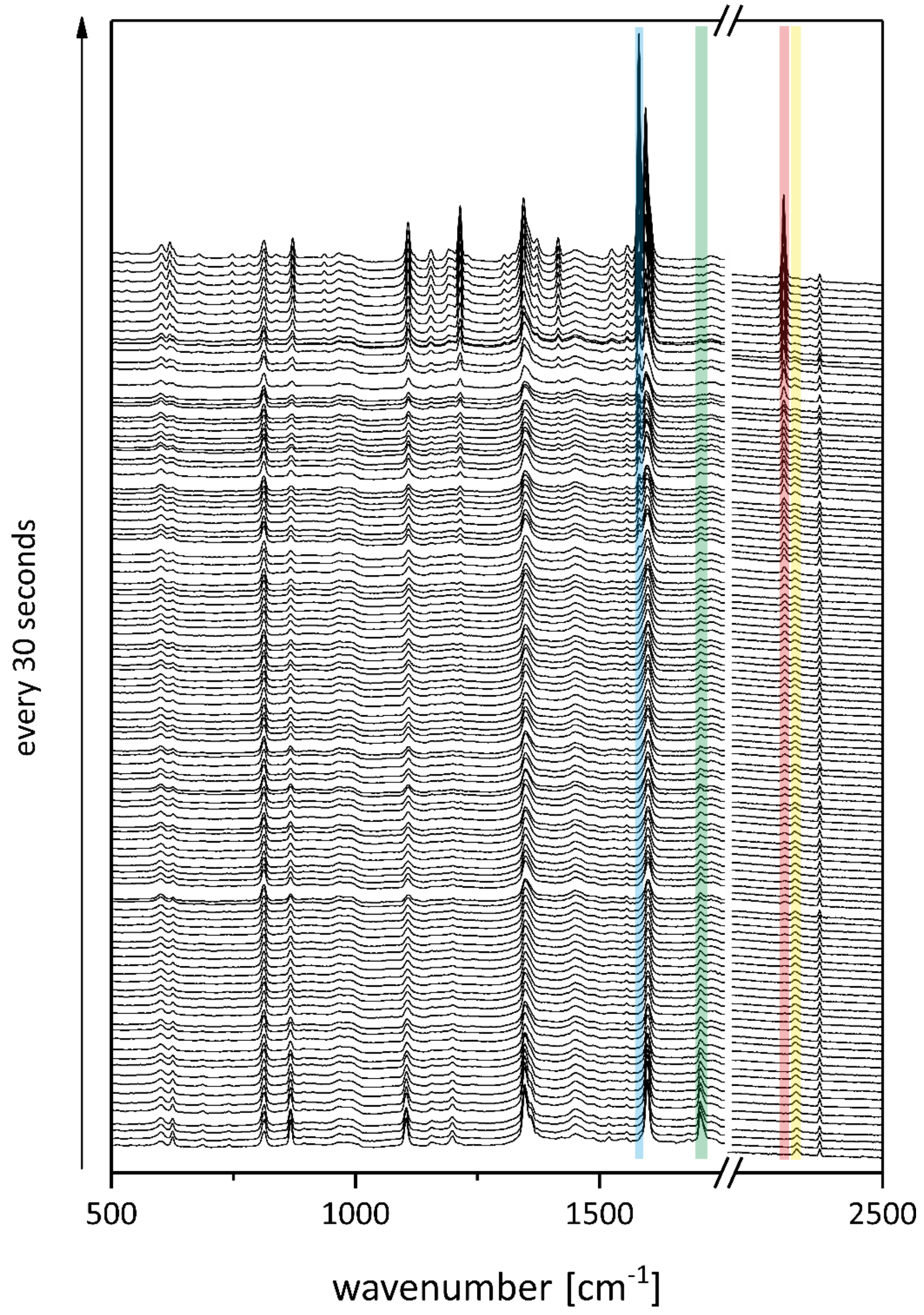

Figure S1: Time-resolved Raman spectra measured simultaneously The progress of the reaction can be detected from the decreasing signal of the C=O-stretching band of **1** at 1706 cm^{-1} (green) and the increasing signal of the C=C-stretching band at 1581 cm^{-1} (blue). The band attributed to the C≡N-stretching shifts from 2266 cm^{-1} (yellow) in **2** to 2233 cm^{-1} (red) in the product.

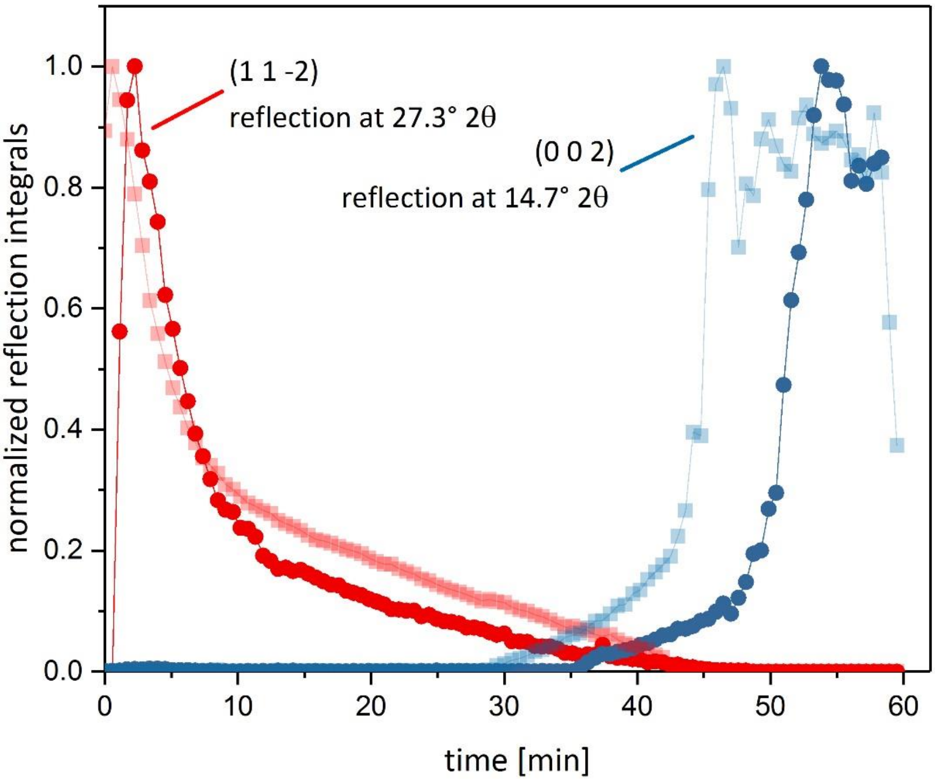

Figure S2: Quantitative evaluation of the PXRD reflections intensities of selected reactant (red (11-2) reflection of **1**) and product reflections (blue (002) reflection of **3**). The PXRD data are representative measurements of repeated sets measured at two different beam times (intensive colour November 2016 and light colour August 2017). The data indicate a high reproducibility of the results. The cause of the reaction can be divided in three steps. First a fast consumption of the reactant 1 is observed. After 11 minutes a decelerated consumption is observed. The formation of the product 3 starts after approx. 35 minutes and is characterized by a slow (until approx. 48 min.) and finally a very fast conversion.
